# *In vitro* susceptibility of 147 international clinical *Mycobacterium abscessus* isolates to epetraborole and comparators by broth microdilution

**DOI:** 10.1093/jac/dkae461

**Published:** 2024-12-31

**Authors:** Minh-Vu H Nguyen, Vinicius Calado Nogueira de Moura, Tiffany R Keepers, Jakko van Ingen, Charles L Daley

**Affiliations:** Division of Mycobacterial and Respiratory Infections, Department of Medicine, National Jewish Health, Denver, CO, USA; Division of Mycobacterial and Respiratory Infections, Department of Medicine, National Jewish Health, Denver, CO, USA; Department of Clinical Microbiology, AN2 Therapeutics, Inc, Menlo Park, CA, USA; Department of Medical Microbiology, Radboud University Medical Center, Nijmegen, The Netherlands; Division of Mycobacterial and Respiratory Infections, Department of Medicine, National Jewish Health, Denver, CO, USA; Division of Infectious Diseases, Department of Medicine, University of Colorado, Aurora, CO, USA; Division of Pulmonary Sciences and Critical Care Medicine, Department of Medicine, University of Colorado, Aurora, CO, USA

## Abstract

**Background:**

*Mycobacterium abscessus* is a highly drug-resistant non-tuberculous mycobacterium (NTM) for which treatment is limited by the lack of active oral antimycobacterials and frequent adverse reactions. Epetraborole is a novel oral, boron-containing antimicrobial that inhibits bacterial leucyl-tRNA synthetase, an essential enzyme in protein synthesis, and has been shown to have anti-*M. abscessus* activity in preclinical studies.

**Objectives:**

To determine epetraborole MIC distribution for 147 recent *M. abscessus* isolates via broth microdilution.

**Methods:**

*M. abscessus* isolates collected in 2021 from the USA (*n* = 122) from pulmonary sources and during 2019–22 predominantly from Europe (*n* = 25) from pulmonary and extrapulmonary sources had MICs determined by broth microdilution according to CLSI guidelines for epetraborole and a panel of 12 other antimycobacterials. Descriptive analyses were done on the MIC values.

**Results:**

Of the 147 *M. abscessus* isolates, 101 were subspecies *abscessus*, 6 were *bolletii* and 40 were *massiliense*. Epetraborole MICs ranged from 0.03 to 0.25 mg/L and were consistent across subspecies. Epetraborole MIC_50_/MIC_90_ for all isolates were 0.06/0.12 mg/L. When stratified by subspecies, amikacin resistance, clarithromycin resistance and morphotype, the MIC_50_/MIC_90_ values remained 0.06/0.12 mg/L.

**Conclusions:**

Epetraborole demonstrated potent *in vitro* activity against *M. abscessus* with MICs from 0.03 to 0.25 mg/L and consistent activity against all subspecies, resistance phenotypes and morphotypes. These data support clinical evaluation of epetraborole as a therapeutic option for *M. abscessus* disease.

## Introduction

Non-tuberculous mycobacteria (NTM) cause opportunistic infections in susceptible hosts. Among rapidly growing NTM species causing human disease, *Mycobacterium abscessus*—consisting of subspecies *abscessus*, *bolletii* and *massiliense*—is the most common and most drug resistant.^1^ Treatment is limited by a dearth of effective, oral options and frequent, significant adverse effects with commonly used agents.^2^ These factors and an 8 year all-cause mortality as high as 44.8%^3^ stress the need for new, effective and tolerable oral antimycobacterials against *M. abscessus*.

Epetraborole is an orally bioavailable boron-containing antimicrobial that inhibits bacterial leucyl-tRNA synthetase, an essential enzyme in protein synthesis.^4,5^ Epetraborole was shown to be efficacious against *M. abscessus in vivo* in zebrafish and murine models.^6,7^ Two small *in vitro* studies demonstrated low epetraborole MICs for *M. abscessus* ranging from 0.014 to 0.11 mg/L^7^ and from 0.28 to 0.56 µM,^4^ respectively. However, these studies each tested ≤13 *M. abscessus* isolates, which were either reference strains or clinical isolates from Asia or France, and testing methods did not adhere to the CLSI guidelines for *in vitro* susceptibility testing of NTM.^8,9^

A larger sample of clinical isolates, with MICs determined according to the CLSI standards, is needed to better describe and validate the *in vitro* activity of epetraborole against *M. abscessus*. Therefore, we evaluated the *in vitro* susceptibility to epetraborole of 147 recent clinical *M. abscessus* isolates by CLSI-adherent broth microdilution.

## Materials and methods

### Acquisition of isolates

A total of 147 isolates were studied. These included 122 clinical *M. abscessus* isolates from pulmonary sources collected in 2021 in the USA and stored at the National Jewish Health Clinical Mycobacteriology Laboratory (Denver, Colorado, USA). The remaining 25 randomly selected clinical isolates were acquired from Radboud University Medical Center (Nijmegen, the Netherlands) but originated from various countries: 13 from the Netherlands, 2 Brazil, 2 Germany, 1 Belgium, 1 Croatia, 1 India, 1 North Macedonia, 1 the Philippines, 1 Russia and 1 Surinam. Collected during 2019–22, they consisted of 20 pulmonary and 5 extrapulmonary sources: 1 bone, 1 abdominal abscess, 1 blood, 1 ear tissue and 1 lymph node tissue.

### Species/subspecies identification and morphology determination of isolates

The organisms were isolated on Middlebrook 7H11 agar (Hardy Diagnostics, Santa Maria, CA, USA). Isolates from National Jewish Health were identified to the subspecies level by GenoType NTM-DR Version 1.0 line probe (HAIN Lifescience, Nehren, Germany)^10,11^ or laboratory-developed Sanger sequencing using primers that target the 16S rRNA and *rpoB* regions.^1^ Isolates from Radboud University Medical Center were identified to the subspecies level by WGS.^12^ Colony morphology was determined as either smooth or rough by macroscopic examination. For isolates exhibiting both morphotypes, the predominant morphotype was re-isolated and selected for susceptibility testing.

### Susceptibility testing

Susceptibility testing was performed in accordance with CLSI guidelines^8,9^ using custom frozen broth microdilution panels for 13 antimycobacterials (Thermo Fisher Scientific, Waltham, MA, USA): epetraborole, clarithromycin, amikacin, imipenem, linezolid, moxifloxacin, cefoxitin, doxycycline, tobramycin, clofazimine, minocycline, tigecycline and rifabutin. Pure cultures of *M. abscessus* isolates were grown on Middlebrook 7H11 agar and incubated at 30°C for 5–7 days. A 0.5 McFarland suspension was created from scraping the growth from the agar and mixing in a tube with sterile water and 3 mm borosilicate beads. The inoculum was prepared by transferring 1 mL of the 0.5 McFarland suspension into 29 mL of sterile water to create a final inoculum of 5 × 10^5^ cfu/mL. Each 96-well plate containing antimycobacterials in CAMHB was then inoculated using a 95-pin inoculator assembly (Caplugs Evergreen, Buffalo, NY, USA) and incubated at 30°C. Isolates were tested over five batches using the same lot of frozen panels. Each testing batch included the *Mycobacterium peregrinum* ATCC 700686 strain for quality control (QC), with the first two batches also including *Staphylococcus aureus* ATCC 29213, *Enterococcus faecalis* ATCC 29212 and *Pseudomonas aeruginosa* ATCC 27853 for QC. The MIC of each antimycobacterial was read after 3–5 days of incubation at the lowest concentration of antimycobacterial without visible growth. Constitutive macrolide resistance was determined based on the MIC of clarithromycin read at 3–5 days. Isolates with a clarithromycin MIC of <16 mg/L at 3–5 days were incubated longer and read again at 14 days for phenotypic observation of inducible macrolide resistance. The MIC interpretations were based on the latest published CLSI 2023 M24S.^8^

### Statistical and data analyses

Summary statistics, MIC_50_ and MIC_90_ were calculated using Stata software version 16 IC (StataCorp, College Station, TX, USA).

### Ethics

The National Jewish Health Institutional Review Board approved this study (HS-3796). BRANY Institutional Review Board (21-08-595-528) determined that it met criteria for waiver of informed consent [45 CFR 46.116(f)] and criteria to use and disclose protected health information without authorization [45 CFR 164.512(i)2(ii)].

## Results

**Figure 1.**
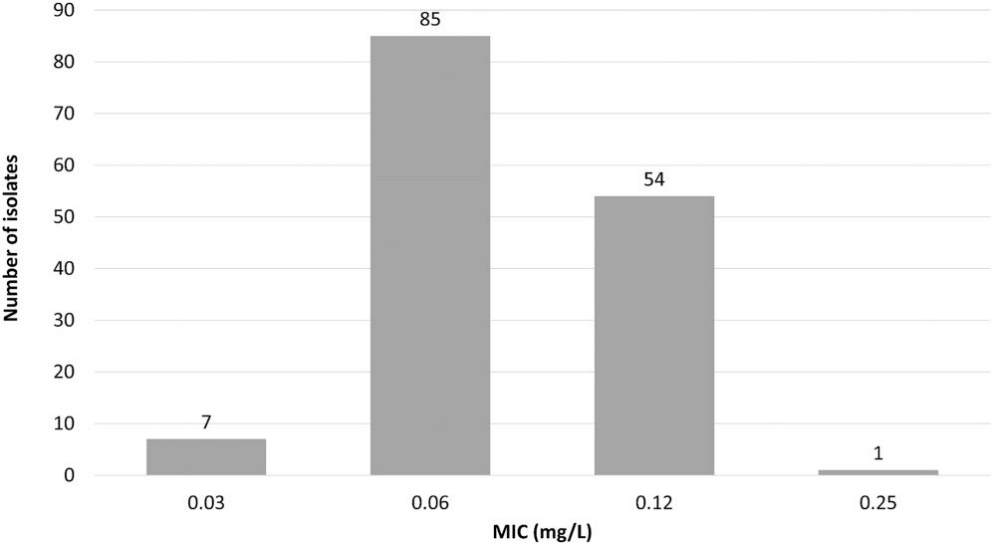
Epetraborole MIC distribution for 147 clinical *M. abscessus* isolates.

Of the 147 isolates in this study, 101 were *M. abscessus* subspecies *abscessus*, 6 were subspecies *bolletii* and 40 were subspecies *massiliense*. Overall, epetraborole MICs ranged from 0.03 to 0.25 mg/L, with 94% of MIC values observed at 0.06 or 0.12 mg/L (Figure 1). For all isolates, the modal MIC was 0.06 mg/L. The MIC_50_ was 0.06 mg/L and the MIC_90_ was 0.12 mg/L for all isolates and for each subspecies (Table 1).

**Table 1.** Epetraborole MIC distribution against *M. abscessus* stratified by subspecies, resistance phenotype and morphotype

	Number (%) of isolates inhibited by specific MIC (mg/L) of:			
	0.03	0.06	0.12	0.25
All (*n* = 147)	7 (4.8)	85 (57.8)	54 (36.7)	1 (0.7)
Subspecies and phenotypes				
*abscessus* (*n* = 101)	3 (3)	58 (57.4)	39 (38.6)	1 (1)
*bolletii* (*n* = 6)	0 (0)	5 (83.3)	1 (16.7)	0 (0)
*massiliense* (*n* = 40)	4 (10)	22 (55)	14 (35)	0 (0)
Amikacin resistant, MIC ≥ 64 mg/L (*n* = 18)	1 (5.6)	7 (61.1)	5 (27.8)	1 (5.6)
Clarithromycin resistant, MIC ≥ 8 mg/L (*n* = 95)	3 (3.2)	53 (55.8)	38 (40.0)	1 (1.1)
Inducible resistance (*n* = 73)	2 (2.7)	43 (58.9)	27 (37.0)	1 (1.4)
Constitutive resistance (*n* = 22)	1 (4.5)	10 (45.5)	12 (50)	0 (0)
Amikacin and clarithromycin resistant (*n* = 10)	1 (10)	5 (50)	3 (30)	1 (10)
Smooth morphology (*n* = 60)	3 (5)	40 (66.7)	17 (28.3)	0 (0)
Rough morphology (*n* = 87)	4 (4.6)	45 (51.7)	37 (42.5)	1 (1.2)

Susceptibility interpretations were according to CLSI (CLSI supplement M24S, 2023).

Table 1 further stratifies the epetraborole MIC distribution by resistance to amikacin and clarithromycin, and by colony morphology. Of the isolates examined, 18 (12.2%) demonstrated resistance to amikacin, with MIC_50_ of 16 mg/L and MIC_90_ of 64 mg/L. Resistance to clarithromycin was observed in 95 isolates (64.6%), with MIC_50_ and MIC_90_ of >32 mg/L. Among the 95 clarithromycin-resistant isolates, 73 (76.8%) displayed inducible resistance, with 67 belonging to subspecies *abscessus* and 6 to *bolletii*. Notably, 10 isolates (6.8%) demonstrated resistance to both amikacin and clarithromycin. Smooth morphology was observed in 60 isolates and rough morphology in 87 isolates. The MIC_50_ and MIC_90_ remained at 0.06 and 0.12 mg/L, respectively, for all subgroups regardless of phenotypic resistance profile or morphology (Table 1). Table S1 (available as Supplementary data at *JAC* Online) shows the susceptibility of the isolates to epetraborole compared with other antimicrobials, along with their MIC_50_ and MIC_90_ values.

Of note, the MIC values of comparator antimicrobials against QC strains were either within the CLSI reference ranges^8^ or one dilution lower than these reference ranges. *M. peregrinum* ATCC 700686 had an epetraborole MIC range of 0.03–0.06 mg/L, falling within the 0.03–0.12 mg/L reference range provided by AN2 Therapeutics, Inc.

## Discussion

This is the first study to examine the *in vitro* efficacy of epetraborole against a large, diverse panel of 147 clinical *M. abscessus* isolates using CLSI-adherent broth microdilution. Epetraborole displayed potent *in vitro* activity compared with other antimycobacterials, with MIC_50_ and MIC_90_ values at least 8-fold lower than the comparators. Furthermore, resistance to clarithromycin and amikacin, the current two most efficacious guideline-recommended antimycobacterials,^2^ did not affect epetraborole *in vitro* activity, suggesting no cross-resistance. Additionally, epetraborole activity was consistent across the three subspecies and between the different colony morphologies. Its narrow range of MICs and immutable MIC_50_ and MIC_90_ across subgroups suggest that epetraborole is a promising novel antimycobacterial against *M. abscessus*.

Although the *in vitro* methodologies by Ganapathy *et al.*^4^ and Sullivan *et al.*^7^ did not use CLSI guidelines and CAMHB, their published epetraborole MIC values were comparable to the epetraborole MIC values obtained in this study. Ganapathy *et al.*^13^ tested 13 *M. abscessus* isolates, which were either reference strains or clinical isolates obtained in Asia, and observed MIC values ranging from 0.28 to 0.56 µM (equivalent to 0.077–0.15 mg/L). Sullivan *et al*.^7^ tested eight reference strains and observed MIC values ranging from 0.014 to 0.11 mg/L.

In conclusion, epetraborole exhibited potent and consistent *in vitro* activity when tested against a large collection of contemporary, international *M. abscessus* isolates, with a narrow MIC range of 0.03–0.25 mg/L, MIC_50_ of 0.06 mg/L and MIC_90_ of 0.12 mg/L. This reliable activity was not affected by subspecies, resistance to amikacin or clarithromycin, or by colony morphology. Therefore, epetraborole holds promise as a novel antimycobacterial against *M. abscessus* and warrants further investigation into its therapeutic potential in patients with *M. abscessus* disease, with pharmacokinetics/pharmacodynamics studies as the logical next steps. These *in vitro* activity data provide a key input into dose selection for disease due to this pathogen.

## Supplementary Material

dkae461_Supplementary_Data

## Transparency declarations

C.L.D. receives research grants/contracts from AN2 Therapeutics, Inc., Bugworks, Insmed, Juvabis and Paratek. He has served on advisory boards for AN2 Therapeutics, Inc., Galapagos, Hyfe, Insmed, Matinas, Nob Hill, Paratek and Spero. He has served as a consultant to Genentech and Pfizer, and he is a member of Data Monitoring Committees for Otsuka and the Bill & Melinda Gates Foundation. J.V.I. has served on the advisory boards for AN2 Therapeutics, Inc. T.R.K. was an employee of AN2 Therapeutics, Inc. All other authors: none to declare.

## Supplementary data

Table S1 is available as Supplementary data at *JAC* Online.
