# Supplementary material for: In vitro susceptibility of 147 international clinical Mycobacterium abscessus isolates to epetraborole and comparators by broth microdilution
Source: J Antimicrob Chemother. 2024 Dec 31;80(3):713–6. doi: 10.1093/jac/dkae461 (PMC11879195; doi:10.1093/jac/dkae461)
Supplement: dkae461_Supplementary_Data [file dkae461_supplementary_data.docx]

| **Table S1.** Epetraborole and comparator antimicrobial minimal inhibitory concentration summary statistics and susceptibility interpretations for *Mycobacterium abscessus* isolates (n = 147) | | | | | | |
| --- | --- | --- | --- | --- | --- | --- |
| **Antimicrobial** | **Minimal Inhibitory Concentration (mg/L)** | | | **%S** | **%I** | **%R** |
|  | **MIC range** | **MIC_50_** | **MIC_90_** |  |  |  |
| Epetraborole | 0.03 - 0.25 | 0.06 | 0.12 | - | - | - |
| Clarithromycin | ≤0.25 - >32 | >32 | >32 | 32 | 3.4 | 64.6 |
| Amikacin | 4 - 64 | 16 | 64 | 53.7 | 34 | 12.2 |
| Imipenem | ≤1 - >32 | 8 | 32 | 29.3 | 56.5 | 14.3 |
| Linezolid | ≤0.5 - >16 | 16 | >16 | 47.6 | 23.1 | 29.3 |
| Moxifloxacin | ≤0.5 - >4 | 4 | >4 | 6.8 | 19 | 74.1 |
| Cefoxitin | 4 -128 | 32 | 64 | 44.9 | 51.7 | 3.4 |
| Doxycycline | 0.25 - >4 | >4 | >4 | 1.4 | 8.8 | 89.8 |
| Tobramycin | 4 - >8 | >8 | >8 | 0 | 0.7 | 99.3 |
| Clofazimine | ≤0.25 - 1 | 0.5 | 1 | - | - | - |
| Minocycline | ≤0.125 - >8 | >8 | >8 | - | - | - |
| Tigecycline | 0.25 -1 | 0.25 | 1 | - | - | - |
| Rifabutin | 0.5 - >4 | >4 | >4 | - | - | - |
| MIC, minimal inhibitory concentration; MIC_50_, minimal inhibitory concentration for 50% of isolates tested; MIC_90_ minimal inhibitory concentration for 90% of isolates tested; %S, percent susceptible; %I, percent intermediate; %R, percent resistant. Susceptibility interpretations were according to CLSI (CLSI supplement M24S, 2023). | | | | | | |
